# Expansion of Armatimonadota through marine sediment sequencing describes two classes with unique ecological roles

**DOI:** 10.1038/s43705-023-00269-x

**Published:** 2023-06-24

**Authors:** John D. Carlton, Marguerite V. Langwig, Xianzhe Gong, Emily J. Aguilar-Pine, Mirna Vázquez-Rosas-Landa, Kiley W. Seitz, Brett J. Baker, Valerie De Anda

**Affiliations:** 1 Department of Marine Science, University of Texas at Austin, Marine Science Institute, Port Aransas, TX USA; 2 Department of Bacteriology, University of Wisconsin-Madison, Madison, WI USA; 3 Department of Integrative Biology, University of Wisconsin-Madison, Madison, WI USA; 4 Institute of Marine Science and Technology, Shandong University, Qingdao, China; 5 Department of Integrative Biology, University of Texas at Austin, Austin, TX USA; 6 Unidad Académica de Ecologia y Biodiversidad Acuática, Instituto de Ciencias del Mar y Limnologia, Universidad Nacional Autónoma de Mexico, Mexico City, Mexico; 7 EMBL Heidelberg, European Molecular Biology Laboratory, Heidelberg, Germany

**Keywords:** Microbial ecology, Environmental microbiology, Next-generation sequencing, Metabolism

## Abstract

Marine sediments comprise one of the largest environments on the planet, and their microbial inhabitants are significant players in global carbon and nutrient cycles. Recent studies using metagenomic techniques have shown the complexity of these communities and identified novel microorganisms from the ocean floor. Here, we obtained 77 metagenome-assembled genomes (MAGs) from the bacterial phylum Armatimonadota in the Guaymas Basin, Gulf of California, and the Bohai Sea, China. These MAGs comprise two previously undescribed classes within Armatimonadota, which we propose naming Hebobacteria and Zipacnadia. They are globally distributed in hypoxic and anoxic environments and are dominant members of deep-sea sediments (up to 1.95% of metagenomic raw reads). The classes described here also have unique metabolic capabilities, possessing pathways to reduce carbon dioxide to acetate via the Wood-Ljungdahl pathway (WLP) and generating energy through the oxidative branch of glycolysis using carbon dioxide as an electron sink, maintaining the redox balance using the WLP. Hebobacteria may also be autotrophic, not previously identified in Armatimonadota. Furthermore, these Armatimonadota may play a role in sulfur and nitrogen cycling, using the intermediate compounds hydroxylamine and sulfite. Description of these MAGs enhances our understanding of diversity and metabolic potential within anoxic habitats worldwide.

## Introduction

Microorganisms outnumber other forms of life and drive biogeochemical cycling on the planet [1]. Ocean floor microbial communities are among the most complex on Earth [2] and play a major role in global carbon and nutrient cycling. However, microbial biodiversity in marine sediments is largely unknown despite its importance. This is due to the difficulty and expense of obtaining these samples and the challenges associated with replicating environmental conditions in a laboratory [3–5]. Metagenomics has provided insights into marine microbial communities by bypassing the need for culturing [6] and has transformed our understanding of biodiversity [1], microbial metabolism [7], and the evolution of life [8]. However, significant gaps remain in our understanding of ocean floor microbes as we have yet to characterize many dominant community members in these systems.

In marine sediments, two microbial guilds responsible for the terminal degradation of organic matter are strictly anaerobic acetogenic bacteria and methanogenic archaea. Both specialized microbial groups, respectively, reduce carbon dioxide (CO_2_) to acetate and methane by the reductive acetyl-CoA pathway (also called the Wood–Ljungdahl pathway, WLP) [9]. Acetogenesis and methanogenesis are processes linked to proton (H^+^) or sodium (Na^+^) ion pumps that drive ATP synthases in the membrane. The processes utilize H_2_ as a major electron donor and CO_2_ as the electron acceptor for energy conservation. In contrast to methanogenesis, acetogenic bacteria can obtain energy through substrate-level phosphorylation and autotrophy, depending on the other metabolic machinery present. Acetogenic bacteria can grow by converting one carbon (C1) substrates (e.g., H_2_CO_2_, CO, and formate) and fermentation substrates (e.g., methoxylated aromatic compounds, sugars, and amino acids, alcohols) to acetate. This metabolic versatility makes acetogenic bacteria an essential player in anaerobic food webs worldwide [10]. Acetogenesis is found in several archaeal lineages [7, 11, 12], but it has historically only been characterized in two bacterial phyla, Firmicutes [13, 14] and Spirochaetes [15]. Recent metabolic reconstructions from environmental genomes indicate acetogenesis also occurs in Chloroflexi [16]. These recent studies highlight that acetogenesis is likely more widespread in the bacterial tree of life than previously thought.

Here, we evaluated the ocean floor microbial diversity of two contrasting marine sediment environments, the Guaymas Basin (GB) and the Bohai Sea (BS). GB is a geologically active region of hydrothermal vents located at a depth of approximately 2000 m in the Gulf of California. In GB, hydrothermal plumes provide an abundance of reduced electron donors for microbial growth, such as H_2_, H_2_S, Fe^2+^, and NH_4_^+^ [17, 18]. GB has high sedimentation rates and organic-rich sediments, and thus can support diverse and active microbial communities [19]. BS is a shallower marine site (average of 18 meters deep) located along China’s northern coast and is connected to several bays and the Yellow Sea. This region is characterized by anthropogenic inputs resulting in high levels of contaminants and eutrophication, primarily driven by agriculture and industry [20, 21].

Our metagenomic characterization of these marine sediment environments allowed us to identify 77 metagenome-assembled genomes (MAGs) belonging to the Armatimonadota phylum (previously known as Candidate division OP10). Armatimonadota representatives were first discovered 20 years ago through 16S rRNA gene surveys in Yellowstone National Park [22, 23]. However, it was not until a decade later that these organisms were designated a new phylum [24]. Difficulties defining this phylum have been caused by a limited number of cultivated Armatimonadota strains, making it challenging to characterize their metabolisms [25, 26] and determine their phylogenetic position. Armatimonadota are known to be aerobic oligotrophs that degrade complex carbon compounds, and no strict anaerobic acetogenic members of this phylum have been yet described. Here, we characterize novel members within the Armatimonadota that are potentially capable of performing acetogenesis, participating in nitrogen and sulfur cycling, and mediating key processes in the anaerobic carbon cycle in marine sediments.

## Materials and methods

### Sample collection

Guaymas Basin (GB) sediments samples (Aceto Balsamico, Megamat19, and Megamat22) were collected during Alvin submarine dives from sediments in the Gulf of California, Mexico, (27°N 0.388, 111°W 24.560) for more details see Langwig, et al. (2021) and Castelle, et al. (2021). Chinese sediment samples were collected from three sites in the Bohai Sea (BS) (BHB10: 38°45.00 N, 118°9.12E; M3: 38°40.03 N, 119°32.51E; and M8: 39°41.34 N, 120°38.98E) during a cruise with the R/V Chuangxin Yi to Bohai Sea in August, 2018 [27]. These sediment samples were collected using a box-sampler. A polyvinyl chloride (PVC) tube with 11 cm internal diameter was inserted into the box-sampler after carefully removing top water to take sediment-core samples. Sub-samples were taken through pre-drilled side-holes with intervals of 2 cm, and frozen at –80 °C.

### Genome sequencing and assembly

Genome sequencing and assembly of GB samples was carried out as described in Langwig, et al. 2021. Briefly, whole community DNA from ≥ 10 g of sediment was extracted using the DNeasy PowerSoil kit (Qiagen, Germantown, Maryland, USA) following the manufacturer’s instructions. The DNA concentrations were quantified with a QUBIT 2.0 fluorometer (Thermo-Fisher, Singapore). Illumina HiSeq 4000 genome sequencing for Guaymas Basin samples was conducted at the Michigan State University RTSF Genomics. Sequences were trimmed and filtered using Sickle v1.33, and assembly was performed using IDBA-UD v1.0.9 More details were described by Langwig et al. [28].

Genome sequencing and assembly for the BS samples is described previously in Gong et al. [29]. Whole community DNA from ≥1 g of sediment was extracted using the DNeasy PowerSoil kit (Qiagen, Germantown, Maryland, USA). DNA concentration was measured using the Qubit® dsDNA Assay Kit in Qubit® 2.0 Fluorometer (Life Technologies, CA, USA). OD values between 1.8 and ~2.0 and DNA contents above 1 μg were used to construct the library. A total of 1 μg DNA per sample was used as input material for the DNA preparations. Sequencing libraries were generated using NEBNext® Ultra™ DNA Library Prep Kit for Illumina (NE, USA) following the manufacturer’s recommendations, and index codes were added to attribute sequences to each sample. Briefly, the DNA sample was fragmented by sonication to a size of 350 bp, then DNA fragments were end-polished, A-tailed, and ligated with the full-length adaptor for Illumina sequencing with further PCR amplification. Finally, PCR products were purified (AMPure XP system), and libraries were analyzed for size distribution by Agilent2100 Bioanalyzer and quantified using real-time PCR before sequencing. DNA from the BS samples were sequenced with an Illumina HiSeq X™ Ten platform at Tianjin Novogene Bioinformatic Technology Co., Ltd (Tianjin, China). BS-derived sequences were trimmed and quality controlled using Sickle v1.33, and assembly was performed using IDBA-UD v1.1.3.

### Genome binning

Binning of individual GB assemblies, only scaffolds >2000 bp, was performed from dives using Concoct v.0.4.0 [30] and Metabat v2.12.1 [31]. Concoct was used with default settings, and Metabat was run with the following parameters: –minCVSum 0 --saveCls -d -v --minCV 0.1 -m 2000. Results from these two binning tools were combined using DAS Tool v1.0 using default settings. CheckM v1.0.11 was used to determine MAG completeness and contamination. Genomes were only analyzed further if they were more than 50% complete and less than 10% contamination. In a previous analysis conducted in Baker, Appler, and Gong (2021) [5], 69 MAGs were identified as a potentially novel phyla (CP9) and included in this research.

The genome binning procedures for BS samples were similar to those for the GB samples. Scaffolds under 2000 bp were removed. Binning was carried out by DAS Tool, Concoct v0.4.0 [30] Metabat v2.12.1 [31], and MaxBin v2.2.7 [32]. The first three binning tools used the same settings as for GB samples. MaxBin v2.2.7 was run with default settings. CheckM v1.1.2 was used to determine MAG completeness and contamination. Genomes were only analyzed further if they were more than 50% complete and contained less than 10% contamination. 8 MAGs were identified as candidates within CP9 in a phylogeny previously described in Gong et al., 2022 [29] and included in further analyses. In total, 77 MAGs were obtained from deep-sea GB and BS coastal sediments. Genome statistics are shown in Supplementary Table 1. The estimated complete genome size was calculated by using a ratio of the recovered MAG size and single copy marker gene completeness based on CheckM.

### Relative Abundance

MAG relative abundance was calculated using MetaGaia (https://github.com/valdeanda/MetaGaia). Briefly, we used the files_prep.py script to link the depth file for each scaffold in the assembly, the total number of raw reads, the length of each bin, and the sampling site of each bin. The output files were reformatted, and the abundance was calculated using the bin_abundancy.py script with the parameter: -n 109.

### Phylogenetic analyses

A set of 318 publicly available genomes was downloaded from NCBI (late 2020 and early 2021) to better resolve the phylogenetic relationships of the MAGs described in this study (Supplementary Table 2). Phylogenetic markers were then extracted from the GB, BS, and reference genomes using phylosift (v1.0.1) [33], with the ‘phylosift search’ and ‘phylosift align’ functions. The genomes were aligned using Geneious Prime v11.0.4 + 11  using MUSCLE v5.1 and MAFFT v7.490 with default settings and were then masked (with at least 50% gaps). A phylogenetic tree was generated through a maximum likeliness-based approach using IQTree v2.0.3 with 1000 bootstrapping replicates using model LG + F + R10 [34]. The tree was visualized using the Interactive Tree of Life (iToL v5). Barrnap v0.7 [35] was used to extract 16 S rRNA gene sequences from the MAGs in this study. These sequences were then compared to known 16 S rRNA genes by using BLASTn [36], against the Silva database release 132 [37]. The 16 S rRNA gene tree was created using RAxML v7.0.3 with standard parameters in the ARB software package [38]. The amino acid identity (AAI) profile was generated using the CompareM v0.1.1 option aai_wf [39]. MAGs were also classified using GTDB-Tk 2.1.1 (dataset r207v2) [40] (Supplementary Table 1).

### Hierarchical clustering of genomes based on protein domains

Two unsupervised clustering approaches were performed as described in Langwig et al. [28] using MEBS v1.0 [41]. First, MAGs were scanned against the Pfam v3.0 database to obtain a protein presence/absence profile using mebs.pl -comp option, then MAGs were hierarchically clustered with mebs_clust.py using Jaccard distance, Ward variance minimization, and a maximum distance threshold of 0.4 (options –distance –method and –cutoff, respectively). Second, the normalized MEBS scores from the 77 Armatimonadota MAGs were clustered along with 2 107 publicly available genomes described in De Anda et al. 2017, and 319 references described in Supplementary Table 2. The clustering approach was performed with the F_MEBS_cluster.py script implemented in MEBS.

### Metabolic predictions

Gene prediction for individual genomes was performed using Prodigal (v2.6.2) [42]. In addition, predicted genes of individual genomes were characterized using several databases: KofamKOALA [43], Interproscan (v5.31.70) [44], HydDB [45], dbCAN (v2.0.11) [46], and MEBS (v1.1) [41].

Hydrogenases were identified through similar methods as described by Langwig et al. 2021 and De Anda et al. [7]. Briefly, hydrogenases were identified using DIAMOND v0.9.26.127 [47] against the reference hydrogenase database and then filtered to ensure an alignment length cutoff of >40 amino acid residues and a sequence identity >50%. Identified sequences were validated using the HydDB web server [45]. No FeFe- or Fe- hydrogenases were identified. We identified 113 NiFe hydrogenases which were used to construct a phylogenetic tree with a compiled database of known NiFe hydrogenases previously described [48]. The sequences were aligned through Geneious Prime v11.0.4 + 11 using the locally contained MUSCLE v5.1 and MAFFT v7.490 programs. A tree was created of the aligned hydrogenase sequences using IQ-Tree v2.0.3 on model LG + R10 using the same parameters as the 37-marker gene alignments and phylogeny and were visualized in iToL v5.

Annotated proteins from all sources were mapped onto metabolic pathways using the KEGG Mapper tool [49], MetaCyc pathway information [50], and manual curation. Hits for key metabolic marker genes found were verified using BLASTP through the NCBI web server tool.

### Hydroxylamine annotation

To determine potential function of possible hydroxylamine-utilizing proteins (hydroxylamine oxidoreductase, Hao; and hydroxylamine dehydrogenase, Hcp), first we performed a BLASTP search against the NCBI non-redundant database (June 2021) to determine closely related sequences based on protein homology (Identity >80%, Coverage >50%). Then, a reference dataset of each protein sequence was obtained via different databases including 154 reviewed sequences from Uniprot [51] for Hcp references (TIGR01703), and 880 sequences from Interpro through the web server (PF02335) used as Hao references [52]. Both publicly available references and hydroxylamine-like proteins identified in the MAGs sequences were aligned with MUSCLE v3.8.31 and Mafft v7.310 using default settings and masked (at least 50% gaps) in Geneious Prime 2021.0.3. The phylogenetic trees were generated using IQTree v2.1.4 with 1000 bootstrapping replicates. Model WAG + R10 was used for Hao, containing 867 sequences in the tree. Model Q.pfam+R6 was used for Hcp, with 191 sequences. The trees were visualized using iTOL v5 and refined in Affinity Designer.

### Carbohydrate-active enzymes (CAZyme) and peptidase identification

We used HMMER, DIAMOND, and Hotpep tools within dbCAN v2.0.11 [46] to identify the CAZymes. We also included signalP [53] prediction and PSORT v3.0 [54] subcellular localization search on valid hits. Only CAZymes annotated by at least two tools were  considered valid and characterized with their corresponding subcellular localization. To identify the peptidases, we downloaded the MEROPs database v12.1 [55] and performed DIAMOND searches against all MAGs described in this study. We assigned possible substrates and families to each hit based on the MEROPs IDs. We only kept hits with known possible substrates. Localization searches were conducted in the same manner as described above for CAZymes.

## Results

### Reconstruction of Armatimonadota genomes from marine sediments

Sixty-nine MAGs (previously classified as a novel phylum [5]) were recovered from three hydrothermally impacted sediment cores from GB (described in Langwig-De Anda et al., 2021 [28]). In addition, eight MAGs were obtained from three sediment cores from BS in Bohai Bay and Midline sites (described in Gong et al., 2022 [29]). Based on the presence of single-copy marker genes inferred by CheckM v1.0.11 [56], these 77 MAGs have an average completeness of 77.35% and an average contamination of 3.59%. The MAGs range in size from 1.28 to 7.02 Mbp, with a median of 3.24 Mbp. The estimated average genome size is 3.9 Mbp. In addition, the MAGs have a high GC-content [57] ranging from 55–70% (Supplementary Table 1 and Supplementary Fig. 1).

### Phylogenetic relationship and taxonomic affiliation

Using several phylogenomic approaches (see methods), we determined that the MAGs obtained in this study fall within the Armatimonadota phylum. GTDB-Tk 2.1.1 (dataset r207v2) [40, 58] indicates that most GB and all BS MAGs are within undescribed classes UBA5377 and CAIYQO01, respectively (Supplementary Table 1). Because our UBA5377 MAGs were entirely identified in the Gulf of California/Sea of Cortez, located in Mexico [5], we propose renaming this undescribed class “Zipacnadia”, after the Mayan mythological figure Zipacna who personified the geological activity of the Earth’s crust [59]. As most of our CAIYQO01 MAGs were identified in the Bohai sea, we propose renaming this undescribed class “Hebobacteria”, after the Chinese mythological figure Hebo, who represents the Huang He River, a major input to the Bohai Sea.

**Fig. 1:**
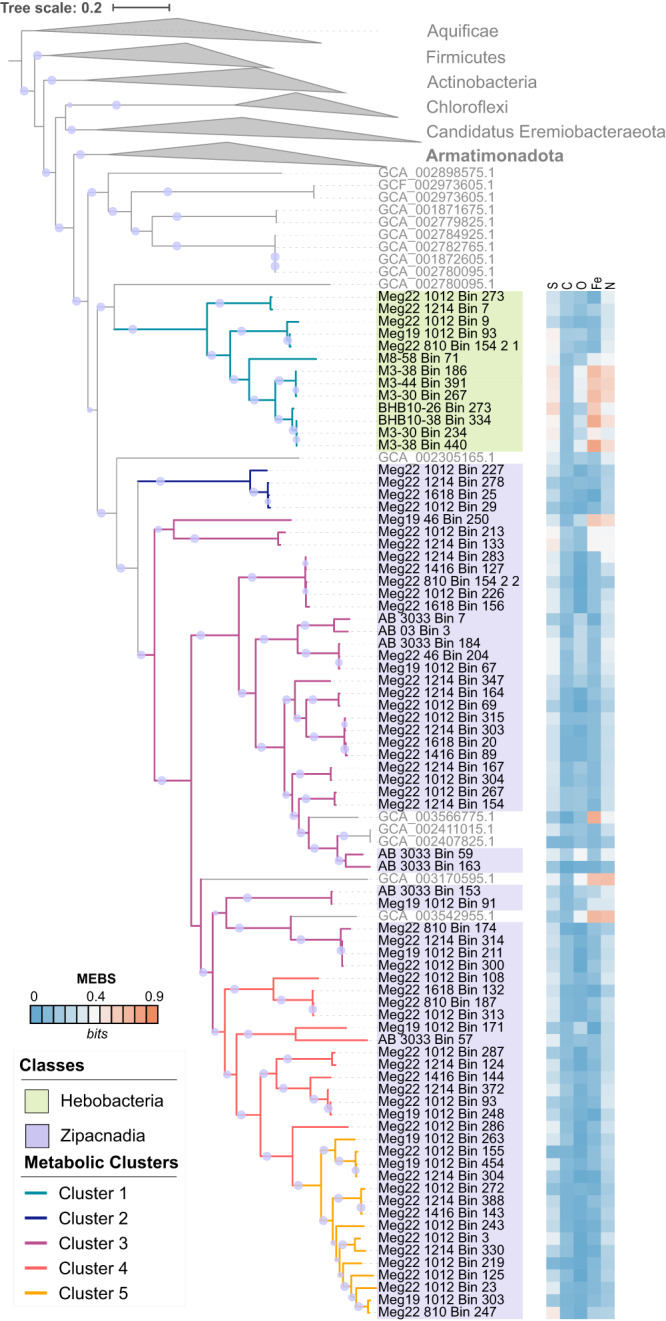
Phylogeny of recovered genomes with cultured and uncultured references, based on 37 conserved marker genes. Bootstrap values over 70% are displayed with the proportional size of circles along internal branches. The two classes are shown to be distinct when compared to all NCBI database available Armatimonadota (100% bootstrap value). The two described classes: Hebobcteria and Zipacnadia are highlighted within the tree in green and purple. Aquificae was used as an outgroup to root the tree. The five metabolic clusters generated based on protein content are shown by the color of tree branches. Scores generated by MEBS are displayed by color blocks directly outside each corresponding branch label. An interactive version of this tree is available online at https://itol.embl.de/shared/2mUVQn1s5SIs8 as “Main Tree”.

**Fig. 2:**
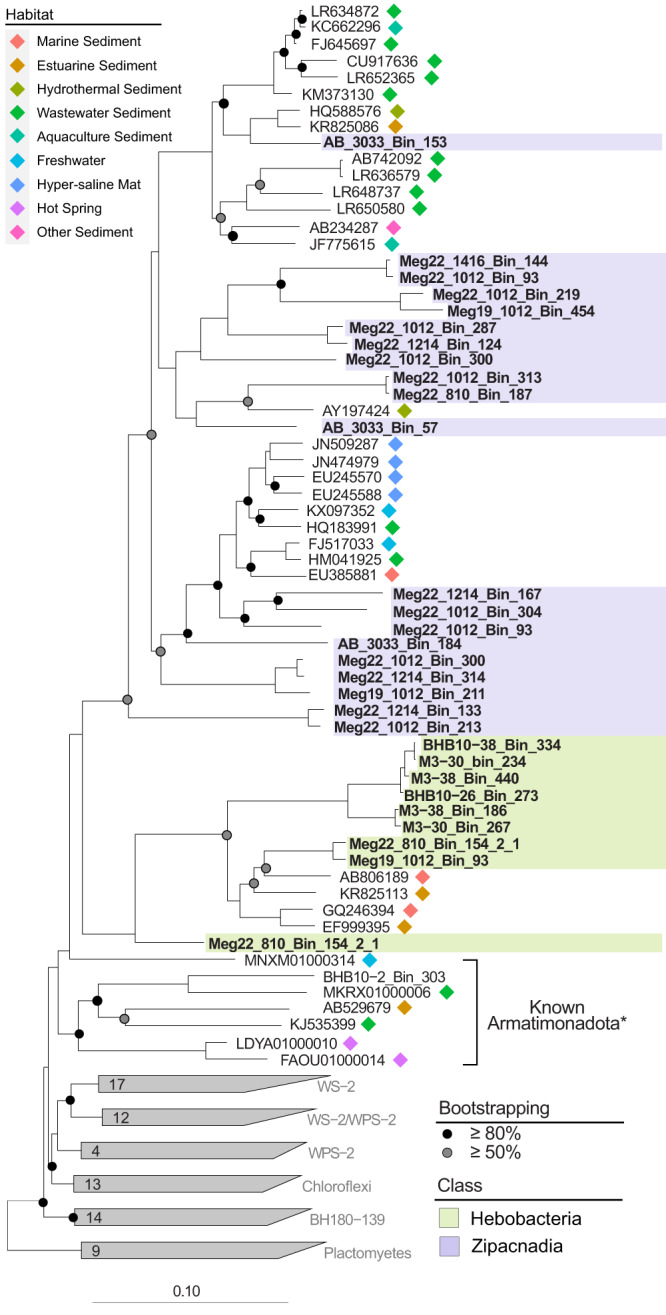
16S rRNA gene phylogeny of recovered MAGs. Two main branches make up the recovered Armatimonadota, the deeper branch contains the shallow sea BS MAGs in Hebobacteria alongside undescribed sequences isolated from the estuary and sea sediments, while the Zipacnadia MAGs are located in the newest branch among other undescribed sequences. Bootstraps are displayed as gray and black circles on internal nodes with greater than 50% and 80% bootstrapping, respectively. Colored markers at the end of undescribed sequences note the environment they were collected from.

**Fig. 3:**
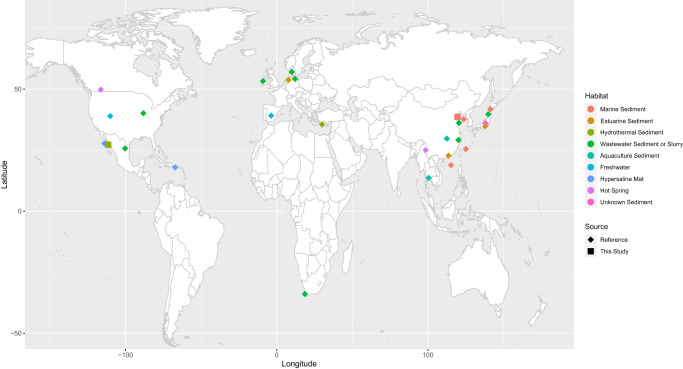
Global map of all Armatimonadota sequences used in 16S rRNA gene phylogeny. Undescribed sequences from the SILVA database (“Reference”) are marked by diamonds, colored to match a particular habitat type. The two locations of MAGs recovered in this study are marked by squares, also colored according to the surrounding habitat. Data collected to create the map is found in Supplementary Table 3.

The 37-marker gene phylogenetic reconstruction, which includes 319 publicly available representatives closely related to Armatimonadota (Firmicutes, Actinobacteria, Chloroflexi, Ca. Eremiobacteraeota) (Supplementary Table 2), confirms the taxonomic relationship of the 77 MAGs within the Armatimonadota phylum (Fig. 1). The 16S rRNA gene phylogeny also revealed 34 unclassified, environmentally derived 16S rRNA genes recovered by past studies closely related to Zipacnadia and Hebobacteria (Fig. 2). These unclassified, publicly available sequences are from globally distributed aquatic sediments in human-derived, freshwater, and marine systems (Fig. 2), suggesting these two understudied classes are globally distributed and may play important ecological roles outside of marine systems (Fig. 3, Supplementary Table 3). A comparison of average amino acid identities (AAI) between our MAGs and 95 publicly available Armatimonadota genomes (Supplementary Table 4) revealed that the Armatimonadota MAGs from deep (GB) and coastal (BS) marine sediments are distinct from previously described Armatimonadota. They share up to 48.56% and 51.46% genome-wide amino acid similarity to one another respectively (Supplementary File 1 and Supplementary Table 4).

### Ecological setting

To estimate the abundance of the marine sediment MAGs (see “Methods”, Supplementary Table 5), we mapped all the metagenomic reads against the genomic assemblies. This revealed that the relative abundance of MAGs from BS was low, averaging 0.0000487% across all sampling locations, though their abundance increased with depth (up to 10 times that of the shallowest samples in the same site). MAGs from GB sediments were obtained from two unique sites. First was Megamat (Alvin cores Meg19 and Meg22 taken within close proximity to each other), an alkane-rich site named for the sizable microbial mat discovered at this location [60]. Second was Aceto balsamico (AB), named after high acetate porewater concentrations at this site, reaching >800 µM. Megamat is low in methane (<1 mM) and high in sulfate (~26 mM), while AB has higher methane concentrations (5.8–8.8 mM) and low sulfate and sulfide (<1 mM). The low abundance of our Armatimonadota MAGs recovered from AB (0.4%), and their high abundance in Megamat (1%) suggests a preferential niche for sulfate-rich environments. In these ecosystems, methanogenesis may not be predominant or is restricted to substrates that cannot be metabolized by sulfate-reducing bacteria, such as methylamines [61].

### Metabolic inference

Several approaches were used to characterize the metabolic capabilities of the 77 Armatimonadota described in this study (see “Methods”). First, we clustered these bacteria based on their protein composition. Hierarchical clustering of the 77 MAGs based on the presence/absence profile of 17,930 protein domains from the Pfam v3.0 database identified five metabolic clusters (Supplementary Table 1). These clusters were consistent with the phylogenetic position of the marine sediment MAGs (Fig. 1).

We also searched the MAGs for involvement in key biogeochemical processes using MEBS (Multigenomic Entropy Based Scores) [41]. MEBS searched for protein sequences involved in nitrogen, iron, oxygen, carbon (primarily methane-related), and sulfur cycling. The normalized entropy scores of the Armatimonadota MAGs, as well as 319 publicly available references (Supplementary Table 2), were compared with a set of previously precomputed scores from 2 107 non-redundant genomes [41] (Supplementary Fig. 2 and Supplementary Table 6). We used three projection methods and four clustering algorithms to analyze the consistency of the clusters. The non-supervised clustering from this analysis suggested that most of the MAGs from deep-sea sediments (58 Zipacnadia and 4 Hebobacteria) are similar and contain fermentative anaerobic pathways (Supplementary Table 6). In contrast, all MAGs obtained from BS sediments (8 Hebobacteria) and some from GB (6 Zipacnadia and 5 Hebobacteria) share protein content with organisms that generate energy by the oxidation or reduction of inorganic sulfur or nitrogen molecules (Supplementary Fig. 3).

To further investigate the metabolic capacity of the novel Aratimonadetes genomes, we compared their predicted proteomes with a combination of functional databases and protein phylogenies. We manually reconstructed the metabolic pathways of the MAGs obtained in this study. These metabolic inferences agree with the MEBS clustering (Supplementary Fig. 3) and suggest that the Armatimonadota MAGs from marine sediment are mainly anaerobic acetogens with different energy production pathways.

### Acetogenic pathways to conserve energy

**Fig. 4:**
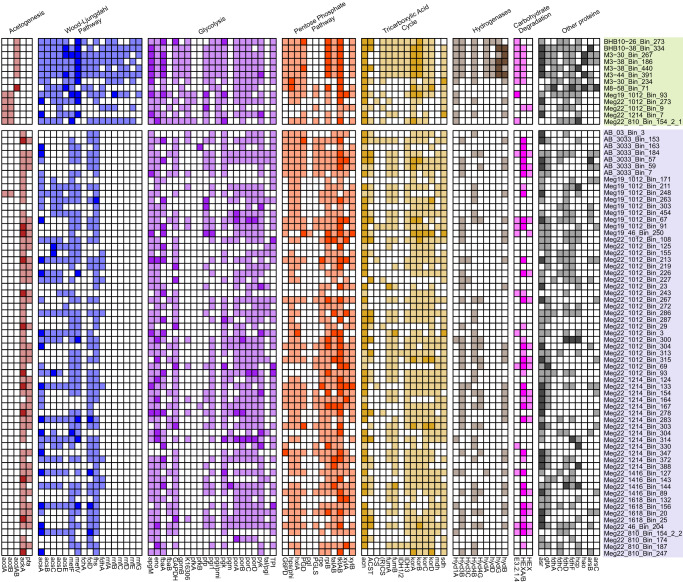
Distribution of key selected metabolic pathways across recovered MAGs. A presence/absence chart of notable metabolic gene annotations among recovered MAGs for Acetogenesis, Wood-Ljungdahl Pathway (including Rnf Complex), electron transport chain, glycolysis, pentose phosphate pathway, tricarboxylic acid cycle, hydrogenases, carbohydrate active enzymes, and other capabilities (such as sulfur and nitrogen utilization). The rows are sorted by class (Hebobacteria on top, Zipacnadia on bottom). Annotations for one copy are shown in the lighter shade of each category, the darker shade corresponds to two or more copies identified in the genome. Complete lists of annotations in the figure can be found in Supplementary Table 7.

**Fig. 5:**
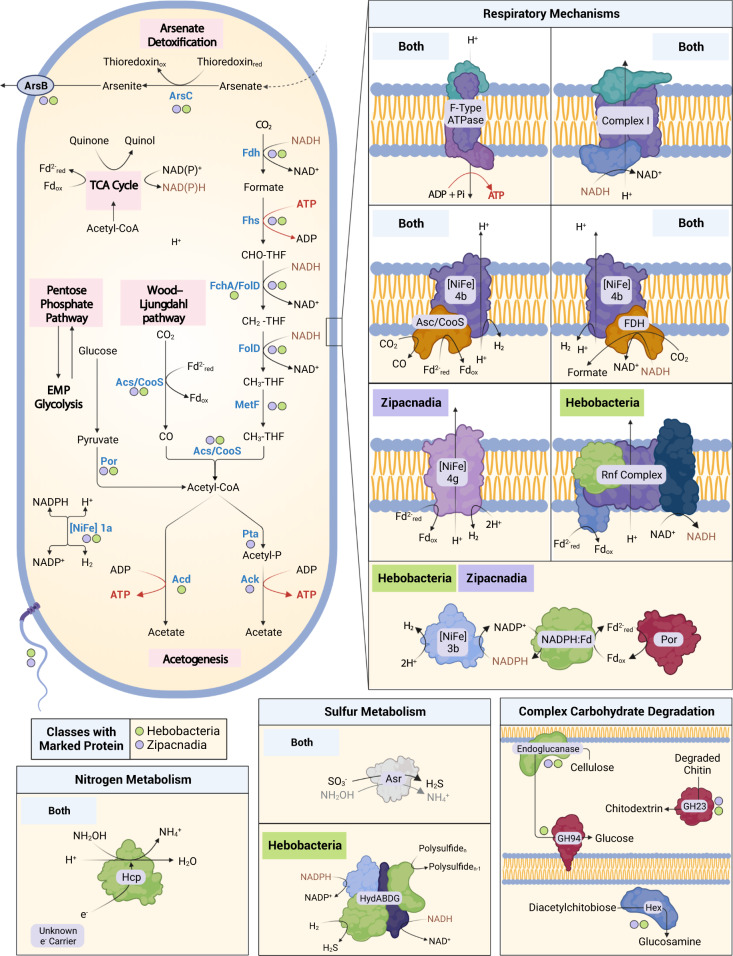
Metabolic potential of Hebobacteria and Zipacnadia. Features of the core metabolism are shown within the central representation of a cell. Pathways or reactions by class are labeled through text and colored dots. Presence in Hebobacteria is noted by a green dot and Zipacnadia by a purple dot. Components of energy generation are shown in the Respiratory Mechanisms box. These collected genomes are unique within Armatimonadota for their likely capability to act on nitrogen and sulfur species. The autotrophic growth allowed by the Rnf Complex in Hebobacteria and Zipacnadia is a key capability of those recovered genomes. Created using BioRender.com.

Most MAGs (48/77) code acetyl-CoA ligase (AcdAB) or both phosphate acetyltransferase (Pta) and acetate kinase (AckA) for acetate production (Fig. 4 and Fig. 5) and ATP generation. Zipacnadia codes the two-step acetate formation pathway with Pta and Ack (Fig. 4), which is common in acetate-forming bacteria [62]. In contrast, Hebobacteria MAGs (Fig. 4) code AcdAB. This indicates these organisms are capable of the reversible one-step conversion of acetyl-CoA to acetate while generating ATP, and allowing for the consumption of acetate in the absence of other substrates [63]. These MAGs also encode an integral membrane multisubunit ferredoxin–NAD+ oxidoreductase, called the Rnf complex, that catalyzes the electron transfer from reduced ferredoxin (Fd^2-^) to NAD+, generating a chemiosmotic gradient for H+ or Na+ [13].

### Carbon fixation and energy production

The WLP is at least partially present in both classes. Only Hebobacteria code a complete WLP (7/13 MAGs), while Zipacnadia have several that are almost complete (Fig. 4). Even though all Zipacnadia and some (6/13) Hebobacteria MAGs do not code for fchA, this enzyme is likely not essential due to the presence of a bifunctional folD in Zipacnadia and Hebobacteria (Supplementary Table 7). Like other acetogenic bacteria, these MAGs can likely oxidize a large variety of organic substrates (e.g., hexoses, pentoses, formate, and formaldehyde) and inorganic substrates such as hydrogen (H_2_) or carbon monoxide (CO), which can be coupled to the reduction of CO_2_.

Some acetogenic bacteria utilize the six-subunit Rnf complex for energy conservation [13]. This complex reduces one NAD^+^ to NADH and simultaneously moves protons or sodium across the cell membrane to generate ATP through ATP synthase. In Hebobacteria MAGs, 2/13 code all six subunits of the Rnf complex, while 7/13 Hebobacteria code at least four subunits. The presence of an Rnf complex is a key indicator of possible autotrophy in Hebobacteria, suggesting these organisms can grow autotrophically via acetogenesis using the WLP (Fig. 5). The combined pathway resembles the previously described autotrophic acetogen isolate *Clostridium ljungdahli­i* [13]. In contrast, Zipacnadia that lacks an Rnf complex, are not likely capable of autotrophic growth and must rely on heterotrophy to drive acetogenesis, usually by consuming glucose [64].

In known autotrophic acetogenic bacteria, electrons from H_2_ and CO_2_ are derived from hydrogen oxidation, catalyzed by hydrogenases such as HydABCD [13]. After searching Armatimonadota MAGs for potential hydrogenases, we identified NiFe group 4b, 1a, 4 g, and 3b hydrogenase. NiFe group 4b hydrogenase can be associated with formate dehydrogenase, carbon monoxide dehydrogenase, and glutamate synthase, which can act as electron-input sources. We identified all of these associated complexes in both classes (Figs. 4 and 5, Supplementary Table 7), suggesting these organisms can pair diverse electron inputs to their hydrogenase [48]. NiFe group 1a hydrogenase is thought to pair H_2_ oxidation with sulfate, metal, or organohalide reduction, and is encoded in 11/13 Hebobacteria and 5/64 Zipacnadia [48]. NiFe group 4g hydrogenase was identified in 16/64 Zipacnadia MAGs, and this group is predicted to utilize reduced ferredoxins from the TCA cycle to synthesize hydrogen and translocate protons [48]. These NiFe group 4g hydrogenases are phylogenetically closely related to NiFe group 4e (Supplementary Fig. 4, red stars). NiFe group 4e hydrogenases are sometimes associated with the Ech complex, an alternative autotrophic acetogenesis mechanism similar to the Rnf complex [48]. However, we found no evidence of an Ech complex in the 77 MAGs described in this study. Finally, Zipacnadia and some (6/13, only in BS) Hebobacteria code for NiFe group 3b hydrogenase, which reversibly couples the oxidation of NADPH to the fermentative evolution of H_2_.

A Nuo complex, better known as complex I in the electron transport chain, is partially coded in 70/77 of the Armatimonadota MAGs (Nuo, Supplementary Table 7), yet no MAGs code for a complete Nuo complex. The presence of a functional complex I in these MAGs could provide an alternative pathway for creating a gradient that could be utilized by an F-type ATP synthase identified in the MAGs (25/77 complete, 52/77 partial, Supplementary Table 7).

### Central carbon metabolism

The Armatimonadota MAGs recovered here are predicted to have largely incomplete TCA cycles that closely resemble other acetate-producing bacteria [50]. Malate dehydrogenase (35/77) replaces malate:quinone oxidoreductase in the Armatimonadota MAGs, and citrate (Re)-synthase (15/77) is more common than citrate synthase (5/77). The presence of citrate (Re)-synthase in these organisms provides evidence for an anaerobic lifestyle because it is inactivated by oxygen [65]. This contrasts with citrate synthase, which functions in comparably oxygen-rich aerobic organisms. Zipacnadia largely lack either citrate synthase (4/64) or citrate (Re)-synthase (5/64), while Hebobacteria lack malate dehydrogenase. Although the TCA cycle is incomplete, reducing power from the TCA cycle can still be generated in the Armatimonadota MAGs using isocitrate dehydrogenase (IDH), 2-oxoglutarate:ferredoxin oxidoreductase (Kor), succinate dehydrogenase (Sdh), and malate dehydrogenase (Mdh) (Fig. 4). Most MAGs recovered in this study also have incomplete pathways for the Embden–Meyerhof–Parnas (EMP), pentose phosphate pathway (PPP), and reverse ribulose monophosphate pathway (Fig. 4, Supplementary Table 7). Over half (42/77) code complete Pyruvate:ferredoxin oxidoreductase (Por), which converts acetyl-CoA to pyruvate, potentially linking the WLP of autotrophic CO_2_ fixation to the TCA cycle. These results suggest that when growing heterotrophically, Armatimonadota (especially Zipacnadia, which lacks autotrophic machinery) may degrade sugars into pyruvate that can be further oxidized to acetate.

### Chemolithotrophy

The presence of anaerobic sulfite reductase (Asr) in most of the MAGs (65 of 77) indicates that these organisms may be capable of reducing sulfite to hydrogen sulfide [66]. The annotated domain from the putative Asr (Supplementary Table 7) suggests this complex may also act on other compounds, such as hydroxylamine (NH_2_OH) or selenium trioxide (SeO_3_) (Fig. 5) [66]. Hebobacteria (6/13, only in BS) also codes at least 3 subunits of HydABGD, a sulfhydrogenase complex that reduces polysulfide to hydrogen sulfide (H_2_S).

Several MAGs contain genes for hydroxylamine-utilizing enzymes (Fig. 4). Hebobacteria (4/13) code hydroxylamine dehydrogenase (Hao) (Supplementary Table 8). However, protein phylogenies suggest these Hao genes are more closely related to cytochrome c552 (Hao-like), which is an electron transporter involved in dissimilatory nitrite reduction to ammonium (Supplementary Fig. 5) [67]. This is in contrast to cytochrome c554 (true Hao), which would reduce hydroxylamine (NH_2_OH) to nitrite (NO_2_^-^) or nitrous oxide (NO). Following these results, Hebobacteria appear to encode only Hao-like genes and not true Hao. The Armatimonadota MAGs also code hydroxylamine dehydrogenase (Hcp) (9/13 Hebobacteria, 21/64 Zipacnadia), which reduces hydroxylamine to ammonium. The phylogeny of Hcp proteins (Supplementary Fig. 6) supports their original annotation as hydroxylamine dehydrogenase. Since we could not identify complete ammonia oxidation or nitrate/ite reduction pathways, the overall function of Hcp in these organisms remains unknown [68].

### Degradation of carbohydrates and proteins

Our analysis of peptidases and carbohydrate-active enzymes (CAZYmes) revealed several metabolic strategies for degrading complex molecules. We detected at least one extracellular peptidase in 10 Hebobacteria and 57 Zipacnadia MAGs. All identified extracellular peptidases are shown in Supplementary Fig. 7, and a comprehensive list of all detected peptidases is in Supplementary Table 9. Genes for extracellular subfamily S8A protease were common across each class, suggesting a subtilisin-like protease is excreted by these organisms to degrade polypeptides [55]. Zipacnadia MAGs encode S8B family peptidases, suggesting they produce a kexin-like protease for alternate polypeptide degradation through Lys-Arg and Arg-Arg cleavage [55]. Family C40 peptidases are present in 11/13 Hebobacteria MAGs, which play a role in cell wall component degradation [55].

CAZYmes were annotated using three methods and were only considered present if they were confirmed by at least two (see methods). Half of the Armatimonadota MAGs (45/77) code CAZYmes, predicted to degrade carbohydrates such as cellulose, chitin, starch, xylan, mannan, pectin, and laminarin (Supplementary Table 10 and Supplementary Fig. 8). Hebobacteria have partial cellulose degradation pathways. Both classes code endoglucanase (Supplementary Table 7) and the cytoplasmic membrane-bound CAZyme family GH94. The pair of enzymes would allow for the degradation of cellulose to cellodextrin and finally to glucose. Most Armatimonadota MAGs (44/77) encode chitin-degrading machinery. For example, both classes code family GH23, suggesting these organisms can initially degrade chitin to chitodextrin (Supplementary Table 10). Inside the cytoplasm, degradation of diacetylchitobiose to glucosamine may be carried out by hexosaminidase based on the presence of HEX/HEXA_B/nagZ (45/77 MAGs).

**Fig. 6:**
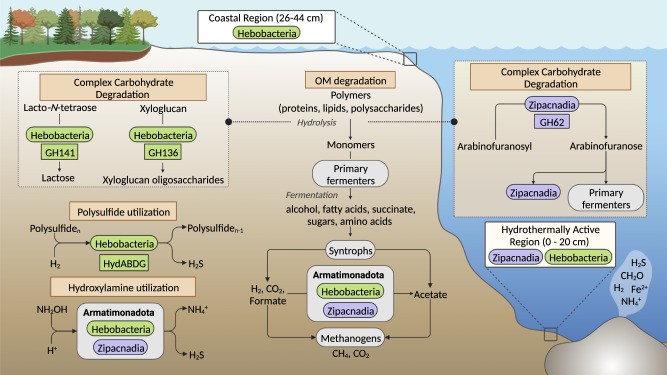
Representation of proposed ecological roles of Hebobacteria and Zipacnadia. Class distinctions are denoted by text and color; all unique environmental contributions are shown with the associated protein colored by the class in which it is present. Eight Hebobacteria MAGs were located in the anoxic sediments of the Bohai Sea with depths of 26–44 cm below the water-sediment interface. Zipacnadia MAGs and 5/13 Hebobacteria were located in the anoxic sediments of the Guaymas Basin with depths of 0–20 cm below the water-sediment interface. All MAGs are anaerobic, functioning primarily on the intermediate compounds sulfite, nitrite, and hydroxylamine provided by sulfate and nitrate reducing microbes in nearby sediments. Organic Matter (OM) includes buried sedimentary carbon metabolized by heterotrophs to produce CO_2_ used directly in the WLP of all MAGs. Both classes likely play a dominant role in supplying usable carbon through acetate secretion and additional degradation of refractory or inorganic matter. Complex carbon degradation is confirmed by the presence of specific extracellular CAZymes (GH62, GH141, GH136), shown here. More distinctions in identified metabolic pathways and capabilities are clearly differentiated between groups in Fig. 4. Created using BioRender.com.

Extracellular CAZYmes were detected in 6/13 Hebobacteria and 39/64 Zipacnadia (Supplementary Table 10). Extracellular hydrolyzing CAZymes were uncommon, with only a few sequences for families GH62, GH121, and GH136. Interestingly, we only identified one extracellular CAZYme in Zipacnadia, GH62, indicating these organisms may degrade arabinofuranosyl to arabinofuranose [69] and supply arabinofuranose to fermenters in the deep sea (Fig. 6). Family GH141 is present in Zipacnadia and Hebobacteria, which allows for the degradation of Lacto-N-tetraose sugars to lactose (Fig. 6) [69]. Family GH136 is present in Hebobacteria, and degrades xyloglucan to smaller oligosaccharides (Fig. 6) [69].

### Arsenate detoxification

Due to its similarity to phosphate, arsenate can freely enter cells through phosphate transporters [70]. A minority of MAGs in both classes possess a detoxification pathway to prevent the buildup of arsenic compounds in their cells. Arsenate within the cell could be transformed into arsenite by an arsenate reductase (ArsC) coded by 16/77 MAGs (Fig. 4). Arsenite can be removed from the cell by an arsenical pump membrane protein (ArsB), present in 21/77 MAGs. A transcriptional regulator responsive to arsenate/arsenite (ArsR) was also found in 53/77 MAGs, likely regulating the expression of the detoxification proteins.

### Environmental interactions and motility

The sampling site of each MAG is the differentiating factor for individual environmental interaction and motility genes. In BS MAGs, we identified a type II bacterial secretion system that participates in biofilm formation. These coastal sediment MAGs code genes for polysaccharide biosynthesis proteins PslF, PslG, and PslH, which support biofilm matrix production. They also code for the adhesion factor PgaC/IcaA, which participates in exopolysaccharide biosynthesis [71–73]. Some GB MAGs (27/69) code PslG, which may help to disperse existing bacteria from biofilms [74]. We identified a type III bacterial secretion system in GB MAGs, identified by the presence of other flagellar structures such as an L ring, P ring, M ring, hook biosynthesis, filament biosynthesis, and other minor basal body components (Supplementary Table 8). Furthermore, the GB MAGs in this study encode motor proteins MotA (40/69) and MotB (37/69), as well as additional genes involved in chemotaxis signal identification and response (Supplementary Table 8). Flagellar structures are common in other deep-sea bacteria, used to avoid temperature and pressure stress [75]. Similarly to previously described Armatimonadota, the MAGs described in this study are likely gram-negative, encoding genes for lipopolysaccharide biosynthetic enzymes, CMP-KDO synthetase, KDO 8-P synthase, 3-deoxy-D-manno-octulosonic-acid transferase, and glucosamine N-acyltransferases (KdsABCD, KdtA, LpxABDL) (Supplementary Table 8).

## Discussion

In this study, we broadly expand the genomic diversity of Armatimonadota bacteria. We describe two classes from marine sediments previously identified as CAIYQO01 and UBA5377 [40], which we propose naming Hebobacteria and Zipacnadia, respectively. Prior to this work, Armatimonadota bacteria were known to be a primarily aerobic heterotroph phylum [25, 76]. The MAGs recovered here expand the diversity of Armatimonadota to include likely obligate anaerobes with chemolithotrophic metabolic potential, some of which may be capable of autotrophic growth. These new Armatimonadota members are broadly distributed worldwide and abundant in deep-sea hydrothermal environments. Zipacnadia genomes are among the most dominant microbial populations in GB sediments (Supplementary Table 5). They are particularly abundant at intermediate depths of anoxic sediments with methane concentrations less than 1 mM, CO_2_ ~10 mM, high sulfate concentrations ~26 mM (similar to ocean water), and temperatures >30 °C. Autotrophic acetogenesis may lead these organisms to compete with methanogenic archaea for hydrogen (H_2_) and carbon dioxide (CO_2_). The large number of Zipacnadia MAGs in Megamat, paired with low methane levels, may suggest that these acetogenic organisms are outcompeting methanogens and thus play a vital role in the final degradation of organic matter in the deep sea. In addition, their flagellar motility may contribute to ecological success in the deep sea, allowing these organisms to navigate the varied microcosms of an active sea floor. Zipacnadia and Hebobacteria (5/13) MAGs are dominant in the deep sea, together making up about 1% of the total microbial diversity in GB, suggesting that understanding their roles is crucial to interpreting the ecology of the ocean floor.

Based on our detailed genomic characterization, the recovered Armatimonadota appear to be acetogenic and fermentative obligate anaerobes. Phylogenetic analyses show that they are divided by the two classes described in this study, though these MAGs appear to be further divided into five separate metabolic clusters based on protein content including undescribed proteins. Our detailed metabolic inferences support the two-class model, with numerous key distinctions in pathways likely to be critical to these bacteria. Hebobacteria MAGs can be distinguished by their potential capacity to grow autotrophically through the WLP and Rnf complex, which augments the proton gradient used by ATP synthase. Zipacnadia, on the other hand, is distinguished by the absence of the Rnf complex, likely relying on a heterotrophic lifestyle that depends on hydrogenases and a complex I-like structure to generate the same gradient. Both classes are likely able to transform inorganic carbon (CO_2_) into acetate, making a carbon pool accessible to other organisms (Fig. 6). Acetate produced by these organisms can become a carbon source, electron donor, or other chemical intermediates for community members.

All Armatimonadota recovered here are predicted to utilize nitrogen and sulfur compounds (Fig. 5). An anaerobic sulfite reductase (Asr) is present in the MAGs, suggesting they play an active role in sulfur cycling. In addition, these MAGs can reduce hydroxylamine (NH_2_OH), an important intermediate in the nitrogen cycle, to ammonium (NH_4_^+^). Hydroxylamine is formed during nitrification and anaerobic ammonium oxidation, and is a precursor of nitrous oxide [77]. However, further research is needed. Little is known about the environmental activity of microorganisms that use hydroxylamine.

Given the breadth of environments where related organisms were identified (16S rRNA gene analysis), it is likely that the MAGs recovered here are important players in anoxic environments globally. Hebobacteria and Zipacnadia appear to fulfill a broad range of ecological roles, vastly expanding the previously known capabilities of Armatimonadota with complex carbohydrate degradation, carbon fixation, nitrogen reduction, and sulfur reduction.

### Proposal of type material

#### Candidatus Hebobacterum abditum

*Candidatus* Hebobacterum abditum (ab.di’tum. L. neut. adj. *abditum*, hidden). A marine sediment taxon reconstructed from environmental sampling, likely autotrophic and capable of unique roles in nitrogen and sulfur cycling. This uncultured lineage is represented by the genome “M3-44_Bin_391”, NCBI BioSample SAMN20205124, recovered from Bohai Sea sediments, and defined as a high-quality metagenome-assembled genome with an estimated completeness of 90.59% and 4.01% contamination, the presence of an incomplete (68%) 23S rRNA gene and 20 distinct tRNAs.

#### Candidatus Zipacnadum vermilionense

*Candidatus* Zipacnadum vermilionense (ver.mi.li.o.nen’se. N.L. neut. adj. *vermilionense*, pertaining to the Vermilion Sea (aka the Gulf of California)). A marine sediment taxon reconstructed from environmental sampling, capable of unique roles in carbon, nitrogen, and sulfur cycling. This uncultured lineage is represented by the genome “AB_3033_Bin_57”, NCBI BioSample SAMN26807170, recovered from Guaymas Basin sediments, and defined as a high-quality metagenome-assembled genome with an estimated completeness of 94.44% and 0.93% contamination, the presence of a complete 16S and 23S rRNA gene, and 20 distinct tRNAs.

#### Candidatus Hebobacterum gen. nov

*Candidatus* Hebobacterum gen. nov. (He.bo.bac.te’rum. Ch. masc. n. *Hebo*, god of the Yellow River; N.L. neut. n. *bacterium*, unicellular microorganism which lack an organized nucleus; N.L. neut. n. *Hebobacterum*, referring to the type genus Hebobacterum). Type species: *Candidatus* Hebobacterum abditum.

#### Candidatus Zipacnadum gen. nov

*Candidatus* Zipacnadum gen. nov. (Zi.pac.na’dum. Sp. masc. n. *Zipacna*, Mayan mythological figure representing the earth’s crust; N.L. neut. n. *bacterium*, unicellular microorganism which lack an organized nucleus; N.L. neut. n. *Zipacnadum*, referring to the type genus Zipacnadum). Type species: *Candidatus* Zipacnadum vermilionense.

#### Descriptions of higher taxonomic ranks

Description of ***Candidatus*** **Hebobacteraceae fam. nov**. (He.bo.bac.te.ra’ce.ae. N.L. neut. n. *Hebobacterum*, referring to the type genus Hebobacterum; *-aceae*, ending to denote a family; N.L. fem. pl. n. *Hebobacteraceae*, the Hebobacterum family). Type genus: Candidatus Hebobacterum.

Description of ***Candidatus*** **Zipacnadum fam. nov**. (Zi.pac.na.da’ce.ae. N.L. neut. n. *Zipacnadum*, referring to the type genus Zipacnadum; *-aceae*, ending to denote a family; N.L. fem. pl. n. *Zipacnadaceae*, the Zipacnadum family). Type genus: Candidatus Zipacnadum.

Description of ***Candidatus*** **Hebobacterales ord. nov**. (He.bo.bac.te.ra’les. N.L. neut. n. *Hebobacterum*, referring to the type genus Hebobacterum; *-ales*, ending to denote an order; N.L. fem. pl. n. *Hebobacterales*, the Hebobacterum order). Type genus: Candidatus Hebobacterum.

Description of ***Candidatus*** **Zipacnadales ord. nov**. (Zi.pac.na.da’les. N.L. neut. n. *Zipacnadum*, referring to the type genus Zipacnadum; *-ales*, ending to denote an order; N.L. fem. pl. n. *Zipacnadales*, the Zipacnadum order). Type genus: Candidatus Zipacnadum.

Description of ***Candidatus*** **Hebobacteria class nov**. (He.bo.bac.te’ri.a. N.L. neut. n. *Hebobacterum*, referring to the type genus Hebobacterum; *-ia*, ending to denote a class; N.L. fem. pl. n. *Hebobacteraceae*, the Hebobacterum class). Type genus: Candidatus Hebobacterum.

Description of ***Candidatus*** **Zipacnadia class nov**. (Zi.pac.na’di.a. N.L. neut. n. *Zipacnadum*, referring to the type genus Zipacnadum; *-ia*, ending to denote a class; N.L. fem. pl. n. *Zipacnadia*, the Zipacnadum class). Type genus: Candidatus Zipacnadum.

## Supplementary information

Description of Supplementary Files

Supplementary Figures

Supplementary File 1

Supplementary Table 1

Supplementary Table 2

Supplementary Table 3

Supplementary Table 4

Supplementary Table 5

Supplementary Table 6

Supplementary Table 7

Supplementary Table 8

Supplementary Table 9

Supplementary Table 10

## Data Availability

All sequence data and sample information are available at NCBI under BioProject ID PRJNA692327 and PRJNA743900 for samples from Guaymas Basin and Bohai Sea, respectively.
