# Supplementary material for: Expansion of Armatimonadota through marine sediment sequencing describes two classes with unique ecological roles
Source: ISME Commun. 2023 Jun 24;3:64. doi: 10.1038/s43705-023-00269-x (PMC10290634; doi:10.1038/s43705-023-00269-x)
Supplement: Supplementary file 1 — Description of Supplementary Files [file 43705_2023_269_MOESM1_ESM.docx]

**Description of Supplementary Files**

**Supplementary Figures**. *Word File.* Supplementary Figures 1-8 with supporting captions.

**Supplementary File 1.** *PDF*. Amino Acid Identity heatmap. Zipacnadia and Hebobacteria are shown under “CP9”. Reference genomes are taken from Supplementary Table 2 and listed in Supplementary Table 4. Red data points mark a more similar amino acid identity between two genomes, suggesting a closer relationship genetically.

**Supplementary Table 1.** *Excel file***.** Genomic statistics and basic information. Zipacnadia and Hebobacteria genomes are listed with bin name, NCBI BioSample ID, genome length, completeness, contamination, estimated complete genome length, predicted number of genes, GC content, sampling location, PFAM cluster (Figure 1), GTDB taxonomy, and proposed class.

**Supplementary Table 2.** *Excel file.* Catalog of publicly available genomes used as references for phylogenetic analysis (Figure 1). Genomes listed on Sheet1 with NCBI sequence accession number, phylum, and NCBI organism name at the time of collection from the database. MEBS scores suggest metabolic capabilities as described in the manuscript. Sheet2 shows all genbank information for the sequences.

**Supplementary Table 3.** *Excel File.* Data used to create the 16S map in Figure 3. Related 16S gene sequences monophyletically grouped with the recovered Armatimonadota MAGs are listed by their sequence identifier. Latitude and longitude were taken according to the most specific information provided by the according BioSample. Original site descriptions are listed alongside the coded habitat type represented in Figure 3.

**Supplementary Table 4.** *Excel file***.** Amino Acid Identity table. The 77 reconstructed genomes and 98 references used in the amino acid identity comparison are shown here in table form. The data was used to create Supplementary File 1. Higher Amino Acid Identity between two genomes suggests a closer genetic relationship.

**Supplementary Table 5.** *Excel file***.** Relative abundance of the collected MAGs across different sample sites in Guaymas Basin (GB) and Bohai Sea (BS). Details of each sample in GB and BS have been described by Langwig et al., 2021 and Gong et al., 2022, respectively. Sheet1 (Bins) lists bin names with their corresponding class and sample site. Sheet2 and Sheet3 show abundance calculations for Bohai Sea and Guaymas Basin samples, respectively. Calculations are described in Methods.

**Supplementary Table 6.** *Excel file.* MEBS Clustering Information of the 77 collected genomes and references from phylogenetic analysis (Supplementary Table 2). Sheet1 (All values) lists all genome names used in clustering with taxonomic information. Reference column notes the source of the genome and cluster referes to which MEBS cluster the genome was placed in. Remaining sheets show the MEBS ooutput for internal program references (Sheet2-MEBS and Sheet3-mebs internal) and phylogenetic references (Sheet4-references).

**Supplementary Table 7.** *Excel file.* Annotations used to create Figures 4 and 5. MAGs are grouped by class in column headers, and metabolic gene identifiers are grouped by pathway for each row. Data values represent the number of hits of each gene are present in the corresponding genome.

**Supplementary Table 8.** *Excel file.* KEGG Annotations of the 77 collected MAGs. KO’s are listed with gene name and description as provided by KEGG database. Genomes are grouped by class across each column. Values represent the number of hits on each KO in each genome.

**Supplementary Table 9**. *Excel File*. Extracellular peptidase presence/absence found through MEROPs search. Sheet1 (MEROPs) lists genomes with all detected extracellular peptidases. Data points represent the number of unique hits for the corresponding identifier in each genome. Sheet2 (MEROPs_data) lists all valid hits individually with localization information.

**Supplementary Table 10**. *Excel File*. CAZymes presence/absence data. Sheet1 (CAZymes_presence) lists genomes by class and all identified CAZyme identifiers. Data points represent the number of unique (different locations) valid (2 or more dbCAN tools) hits for an identifier in each genome. Sheet2 (CAZymes_data) lists each valid hit individually with localization information.
