# Supplementary material for: Expansion of Armatimonadota through marine sediment sequencing describes two classes with unique ecological roles
Source: ISME Commun. 2023 Jun 24;3:64. doi: 10.1038/s43705-023-00269-x (PMC10290634; doi:10.1038/s43705-023-00269-x)
Supplement: Supplementary file 2 — Supplementary Figures [file 43705_2023_269_MOESM2_ESM.docx]

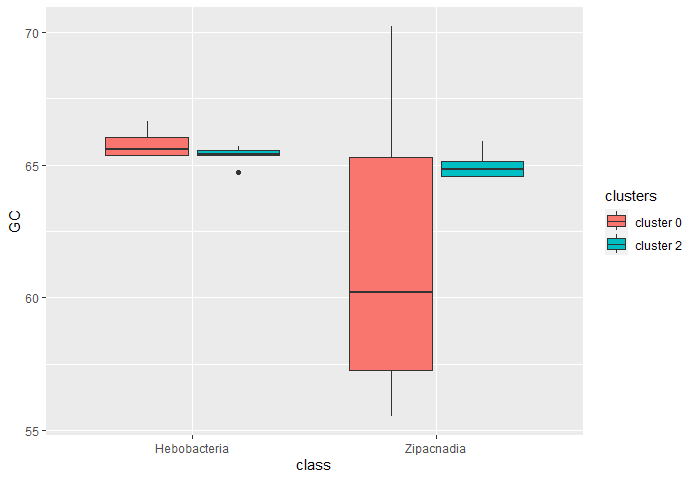


**Supplementary Figure 1.** Distribution of GC-content of the 77 MAGs obtained in this study grouped by their metabolic cluster. The metabolic clusters are shown in Supplementary Figure 3 and detailed in Supplementary Table 6.


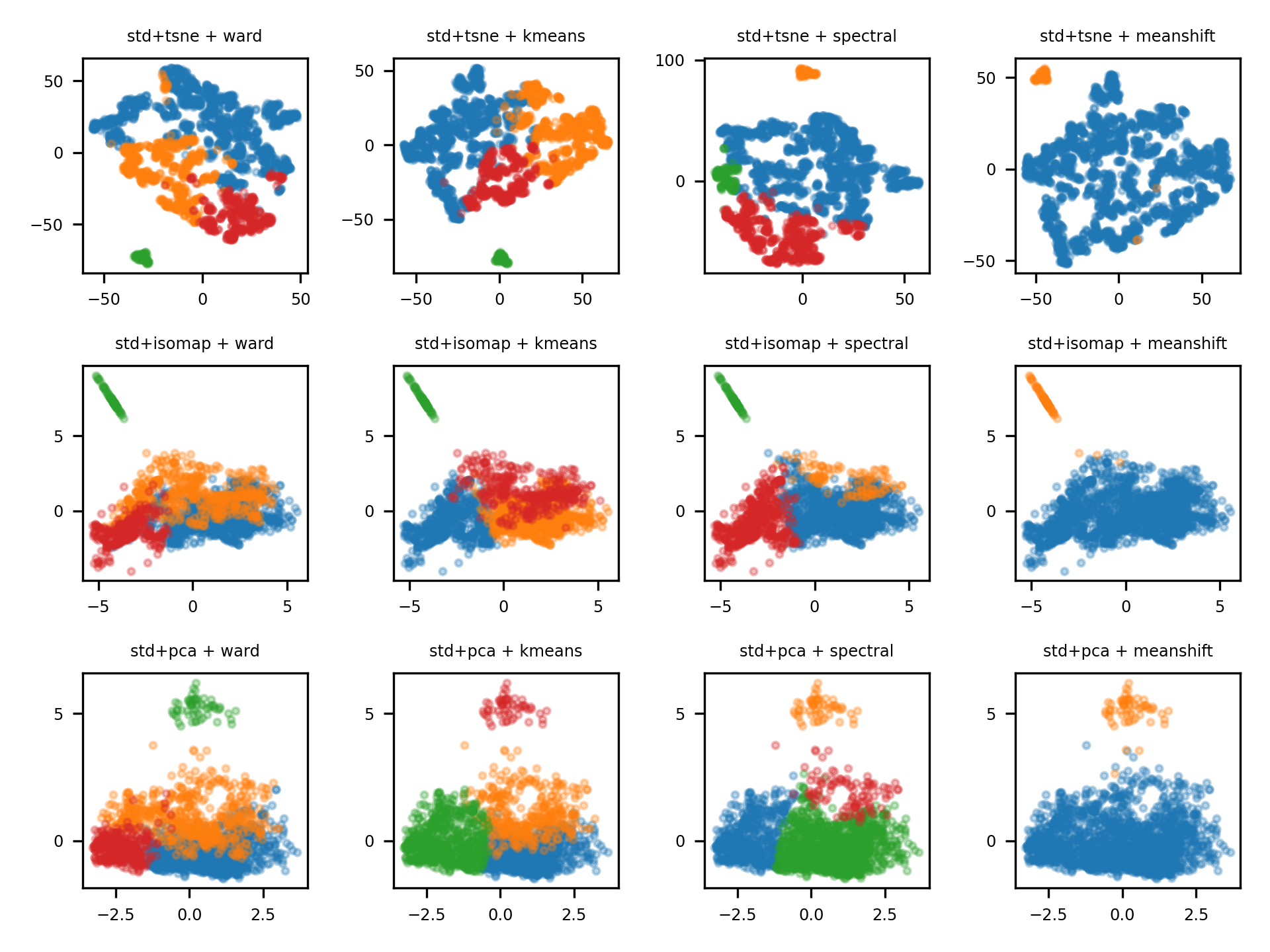


**Supplementary Figure 2**. Clustering of normalized MEBS scores obtained from a genomic-non redundant data set of 2,107 genomes, 319 references described in Supplementary Table 2 and 77 Armatimonadota MAGs described in this study. The figure was obtained with the F_MEBS_cluster.py script implemented in MEBS using the option --all. Details found in https://github.com/bakermicrolab/zipacna/ repository.


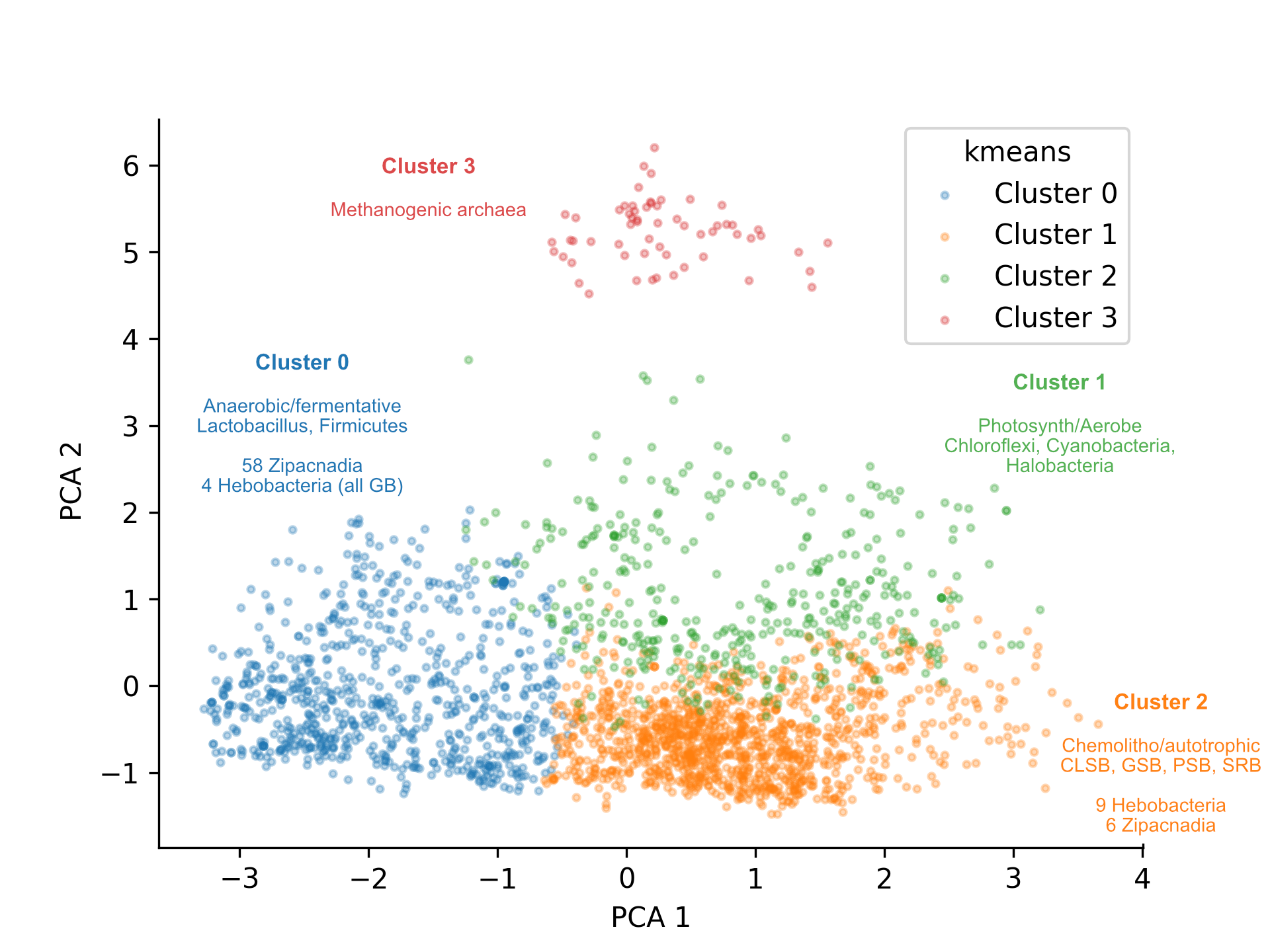


**Supplementary Figure 3**. Clustering of normalized MEBS scores obtained from a genomic-non redundant data set of 2,107 genomes, 319 references described in Supplementary Table 2 and 77 Armatimonadota MAGs described in this study. The figure was obtained with the F_MEBS_cluster.py script implemented in MEBS using the options -p pca and -c kmeans. Details of each group are found in Supplementary Table 6. Abbreviations GB: Guaymas Basin; CLSB: Color-less Sulfur Bacteria; PSB: Purple Sulfur Bacteria; GSB: Green Sulfur Bacteria; SRB: Sulfur reducing bacteria. Details of how to generate the script are found in https://github.com/bakermicrolab/zipacna/ repository.


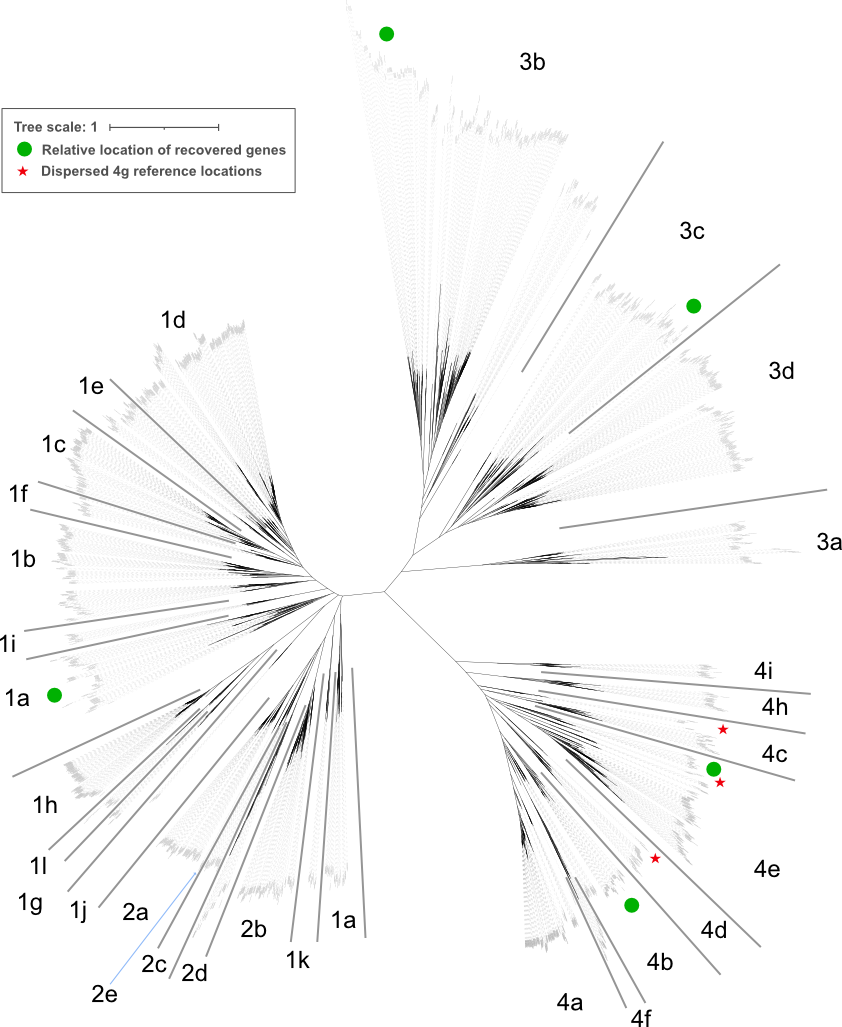


**Supplementary Figure 4.** Phylogenetic tree of 113 detected NiFe hydrogenases in analyzed genomes (via Diamond search and HydDB, see Methods) against the prepared database. Tree is divided by gray bars to separate hydrogenase families. Green circles show the general location of recovered genes in the tree. Red stars show the location of dispersed NiFe 4g hydrogenase sequences. The recovered genes (green circle) noted within the 4e section are most closely related to five disbursed 4g genes. Three group 2e hydrogenases fall on the right side of the group 2a section, noted by a blue arrow and “2e” label. Group 1a hydrogenases are placed in two locations in the tree. The interactive full tree is available online at https://itol.embl.de/shared/2mUVQn1s5SIs8, “Hydrogenase Tree”.

**a)
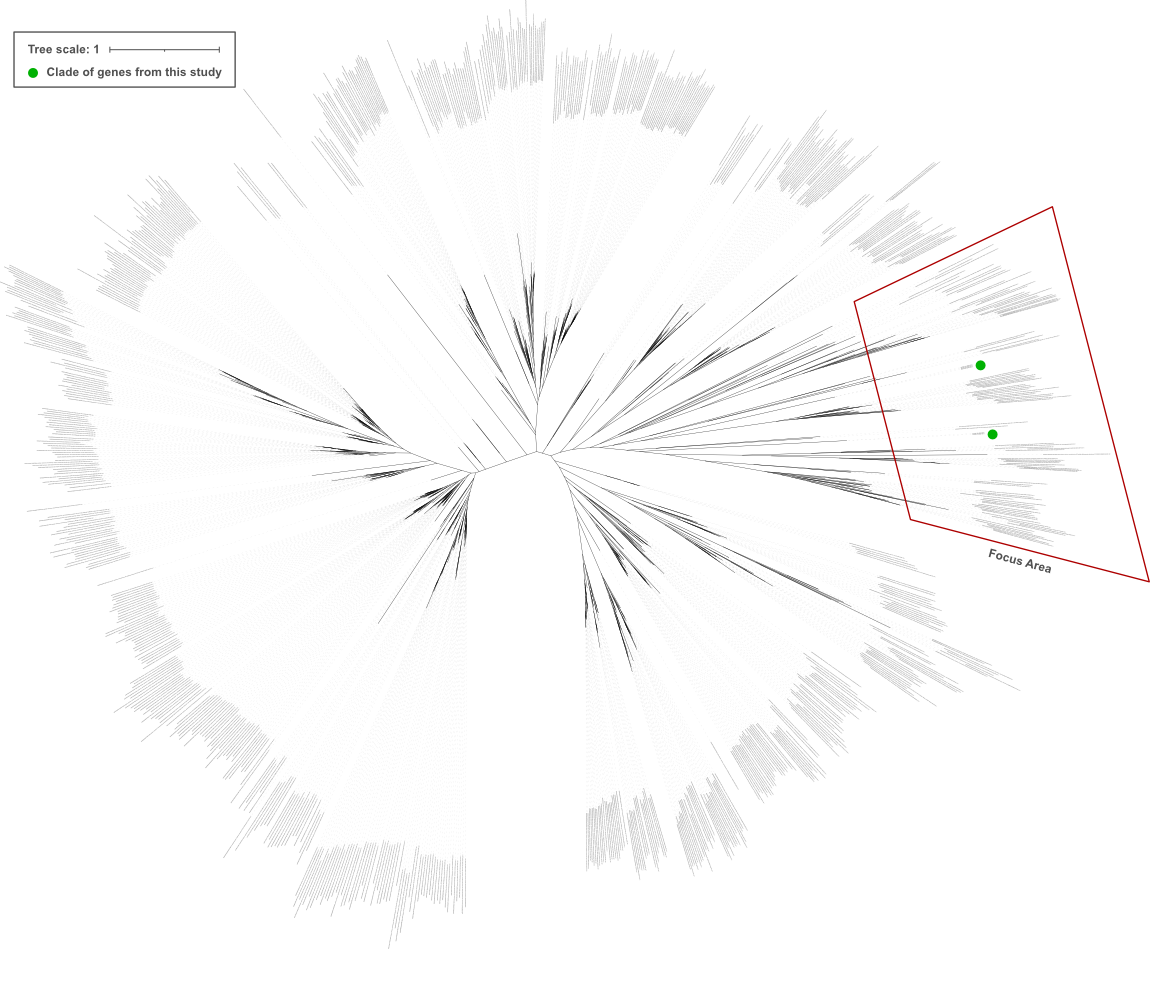
**

**b)
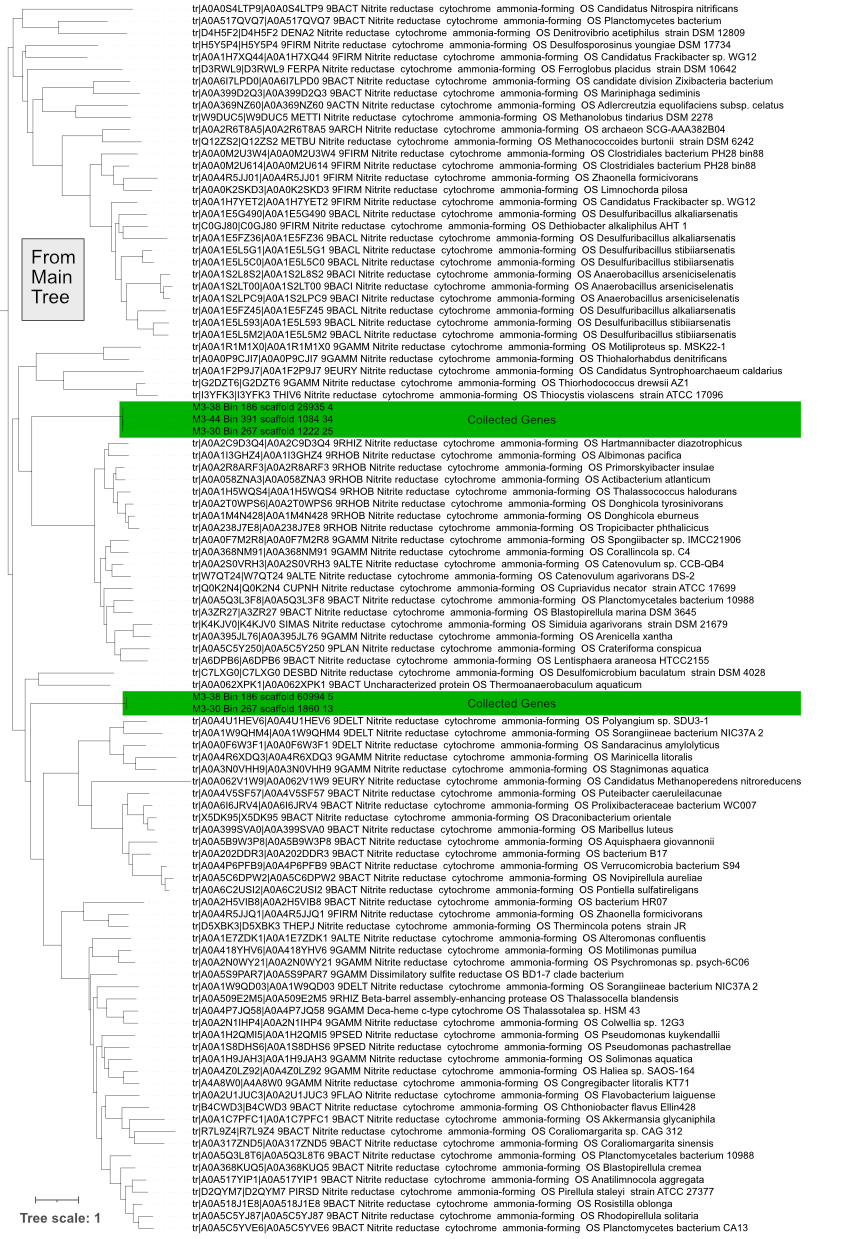
**

**Supplementary Figure 5.** a) Overview of created phylogeny for *hao* genes recovered in this study. Locations of genes collected in this study are marked with a green dot. Focus area outlined in red. Interactive full tree with bootstrap values is available at https://itol.embl.de/shared/2mUVQn1s5SIs8 “Hao Tree”. Tree visualized with the Interactive Tree of Life. b) Focus area of created phylogeny for *hao* genes. Genes collected in this study highlighted green.

a)
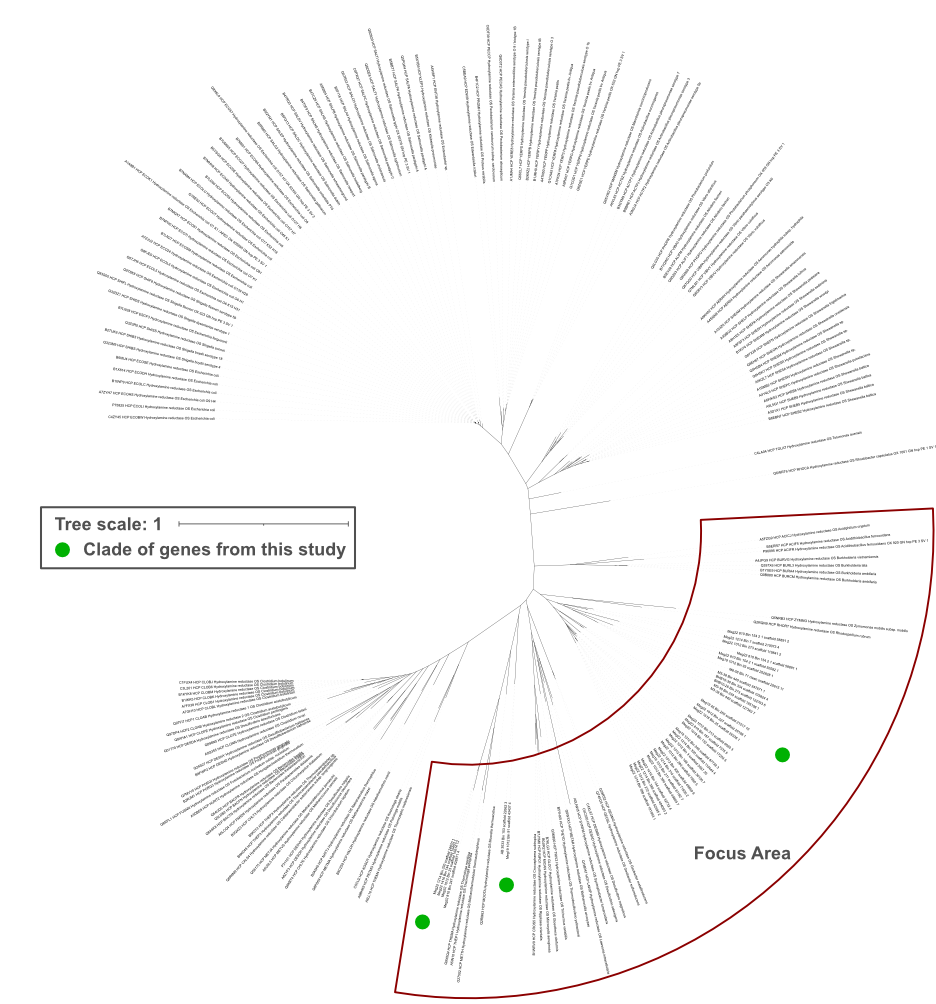


b)
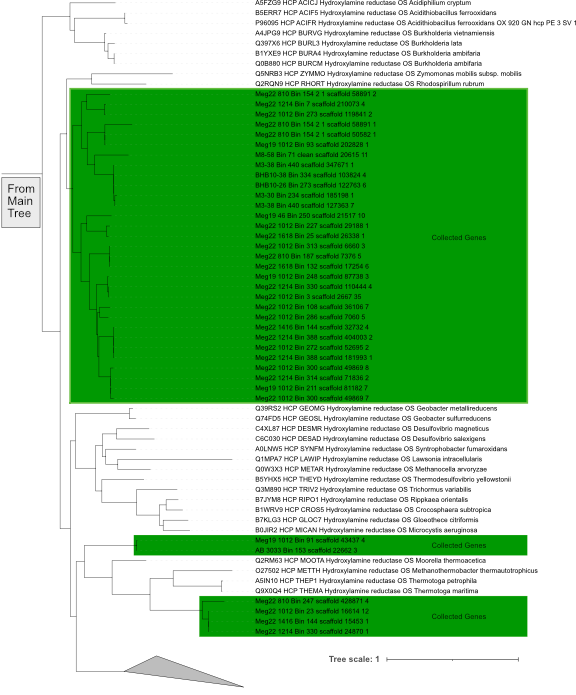


**Supplementary Figure 6.** a) Overview of created phylogeny for *hcp* genes recovered in this study. Locations of genes collected in this study are marked with a green dot. Focus area outlined in red. Interactive full tree with bootstrap values is available at https://itol.embl.de/shared/2mUVQn1s5SIs8 “Hcp Tree”. Tree visualized with the Interactive Tree of Life. b) Focus area of created phylogeny for *hcp* genes. Genes collected in this study highlighted green.

**
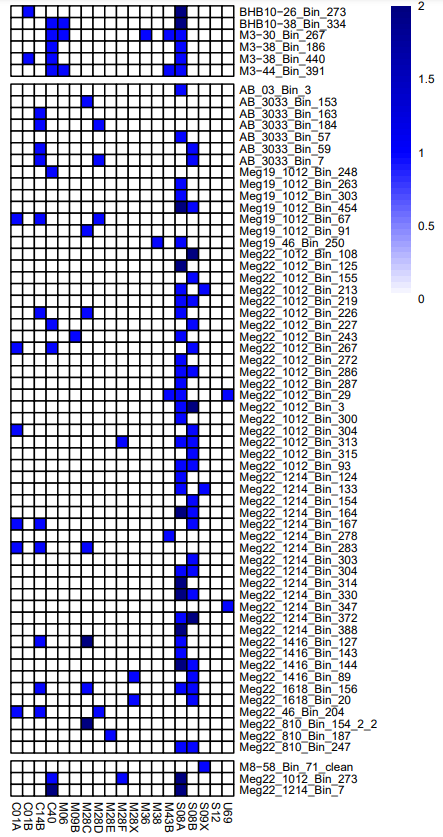
**

**Supplementary Figure 7.** Annotated extracellular proteases encoded by Zipacnadia. Potential localization of the proteases inferred by PSORT. Medium blue is a positive annotation of one copy. Dark blue is a positive annotation of two or more copies. The genomes are separated by class top to bottom, Hebobacteria represented in the top and bottom clustered rows, Zipacnadia in the middle. Data visualized from Supplementary Table 9.


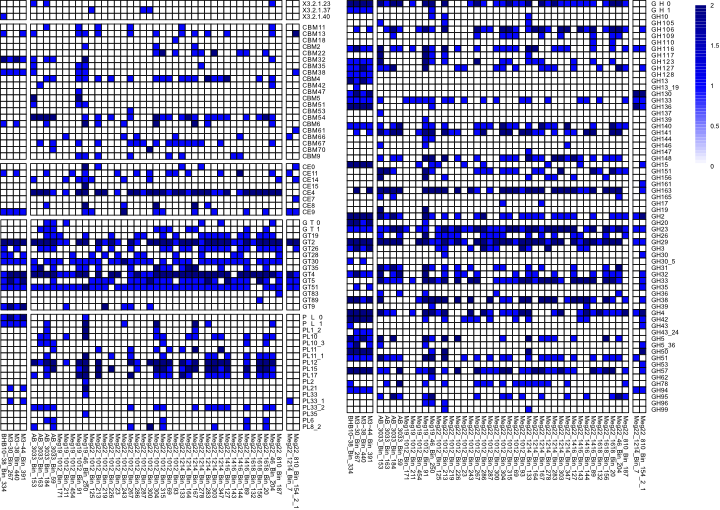


**Supplementary Figure 8.** Annotated carbohydrate active enzymes among recovered genomes. A positive annotation is identified where two of three annotation datasets (Hotpep, HMMER, Diamond) give a positive indication (see Methods). Medium blue is a positive annotation of one copy. Dark blue is a positive annotation of two or more copies. The genomes are separated by class in each chart section, the two outside columns are Hebobacteria, the middle column is Zipacnadia. Data visualized from Supplementary Table 10.
