# Supplementary figures and images for: Expansion of Armatimonadota through marine sediment sequencing describes two classes with unique ecological roles
Source: ISME Commun. 2023 Jun 24;3:64. doi: 10.1038/s43705-023-00269-x (PMC10290634; doi:10.1038/s43705-023-00269-x)

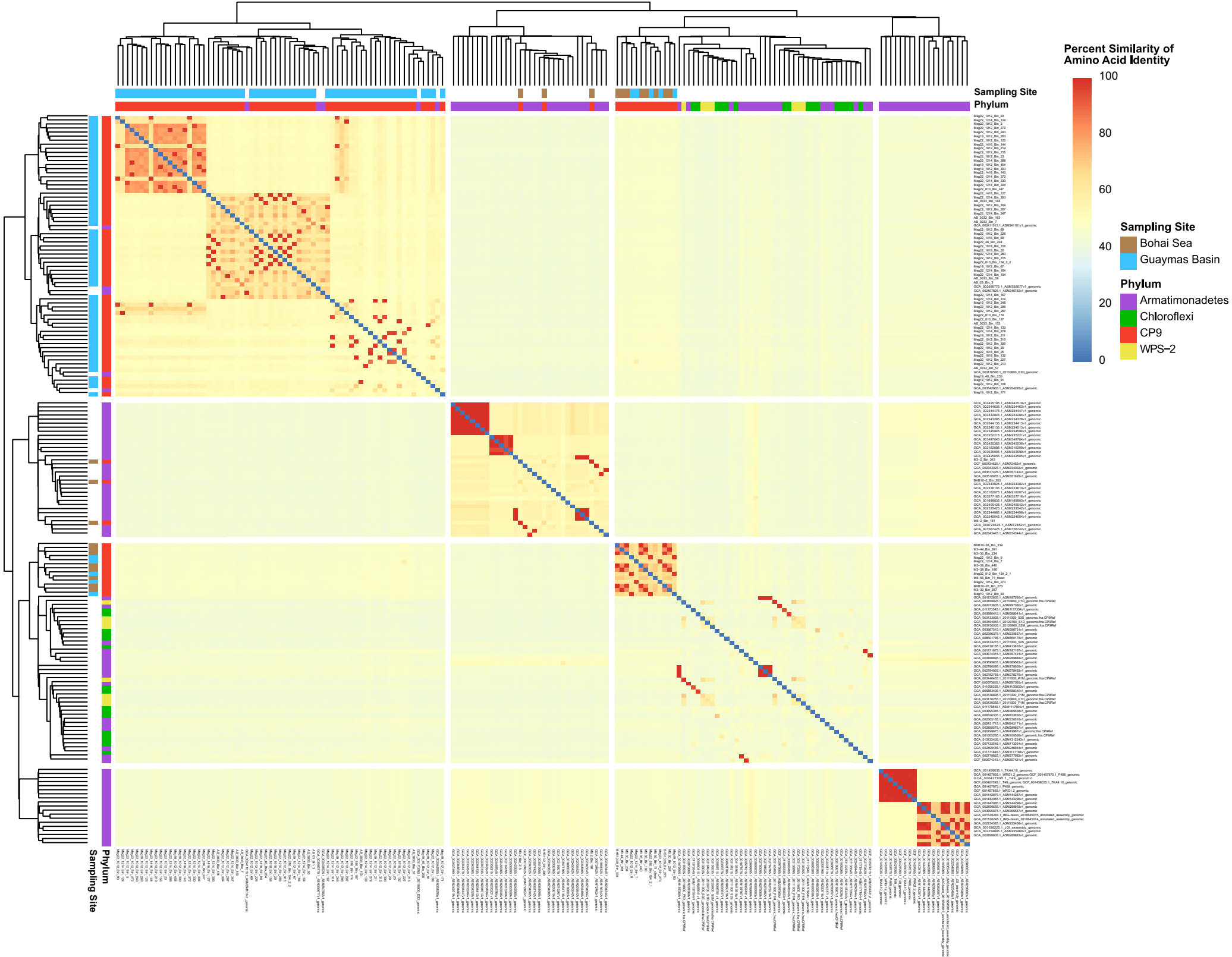

Supplement: Supplementary file 3 — Supplementary File 1 [file 43705_2023_269_MOESM3_ESM.pdf]
